# Feasibility Evaluation of Dried Whole Egg Powder Application in Tadpole (*Lithobates catesbeianus*) Feed: Effects on Growth, Metamorphosis Rate, Lipid Metabolism and Intestinal Flora

**DOI:** 10.3390/ani15040584

**Published:** 2025-02-18

**Authors:** Quan Li, Chuang Shao, Yi Hu, Kaijian Chen, Junzhi Zhang

**Affiliations:** Fisheries College, Hunan Agricultural University, Changsha 410128, China; liquan3149270468@163.com (Q.L.); 13605186516@163.com (C.S.); huyi740322@163.com (Y.H.)

**Keywords:** dried whole egg powder, fishmeal, metamorphosis rate, lipid metabolism, intestinal microbiota, bullfrog tadpole

## Abstract

At present, studies on tadpole nutrition and metabolism are scarce. This study aimed at comparing the influence of two protein sources, fishmeal (FM) and dried whole egg powder (DWEP), on tadpoles from the perspective of growth, the metamorphosis rate, lipid metabolism, antioxidant properties and the intestinal flora. In this experiment, the control diet was set to contain no FM or DWEP. Based on the control diet, 5% and 10% FM or DWEP were included, respectively. The results of the experiment indicated that FM or DWEP inclusion significantly enhanced the growth performance and metamorphosis rate (*p* < 0.05); activated hepatic lipid metabolism, as manifested by enhanced LPL and HL activity; upregulated lipid metabolism-related gene expression (*fasn*, *acc*, *acadl* and *cpt1α*) (*p* < 0.05); and distinctly elevated the activity of SOD, CAT and GPX (*p* < 0.05), suggesting improved antioxidant capabilities (*p* < 0.05). Moreover, the inclusion of FM or DWEP elevated the relative abundance of Actinobacteria and Actinomyces and reduced the relative abundance of Proteobacteria. Unexpectedly, no significant differences were observed between the FM and DWEP groups regarding the above detected indices. This indicates that using DWEP to replace FM is a viable option.

## Simple Summary

This study investigated the feasibility of dried whole egg powder application in tadpole (*Lithobates catesbeianus*) feed, focusing on the effects on their growth, metamorphosis rate, lipid metabolism and intestinal flora. This study indicated that fishmeal (FM) or dried whole egg powder (DWEP) inclusion significantly enhanced the growth performance and metamorphosis rates of tadpoles. FM or DWEP inclusion activated hepatic lipid metabolism. Moreover, the hepatic antioxidant capacity was enhanced by FM or DWEP inclusion, as evidenced by increased SOD, CAT and GPX activity. The supplementation of FM or DWEP stimulates beneficial microbes like Actinobacteria, while suppressing harmful bacteria such as Proteobacteria. These results could provide valuable data for future tadpole feeds.

## 1. Introduction

The bullfrog (*Lithobates catesbeianus*) belongs to the family of omnivorous amphibians and has attracted great attention due to its significant nutritional and economic value [1]. Tadpoles represent the immature stage of bullfrogs, having to undergo metamorphosis to become fully developed. Tadpoles undergo intricate physiological and morphological transformations during this metamorphosis, including tail resorption, lung and leg formation and organ reconstruction, to facilitate adaptation to terrestrial life [2]. The developmental transition is usually modulated by abiotic and biotic factors, such as the ambient temperature, diurnal light cycles [3], dietary resources [4] and predator presence [5]. Presently, due to a lack of specialized compound feeds, the breeding of tadpoles frequently encounters serious challenges, such as a lower metamorphosis rate and higher mortality, consequently causing severe economic losses and environmental pollution. To date, nutritional research on tadpoles has been scarce, and a previous study in our laboratory discovered that tadpoles exhibited the maximum metamorphosis rate when fed a diet with approximately 40% protein and 7% lipids [6].

An adequate protein source is vital for the organism’s development. Fishmeal (FM) is extensively utilized as a premium protein source in aquatic feed, characterized by its excellent nutritional value, digestibility, balanced essential amino acids and functional components. Owing to the continuous decline in global fishery resources, the fishmeal market is facing ongoing supply shortages and instability in sourcing. Aside from FM, alternative animal protein sources such as chicken powder and fermented feather meal can also fulfill the nutritional requirements of aquatic animals [7,8]. However, these alternatives have limitations in terms of production, qualitative stability and cost. In addition, despite being cost-effective, their defects, including anti-nutritional compounds [9] and essential amino acid imbalances, particularly regarding methionine and lysine, limit the large-scale application of many plant protein sources in aquatic feeds [10]. Dried whole egg powder (DWEP) is an economical animal protein source with consistent availability. In addition to high protein content, DWEP also contains a variety of functional substances, such as lecithin and cholesterol, etc., as well as a balanced and rich ratio of amino acids, which may greatly boost animal development. Moreover, DWEP comprises various bioactive chemicals, including immunoglobulin Y, lysozyme, carotenoids and others, which have vital functions in bolstering the immune system and alleviating oxidative stress [11,12]. Investigations into dietary supplementation with DWEP in broiler chickens have revealed significant biological enhancements, including augmented immune response modulation and optimized growth parameters. However, aquatic animals have been the subject of very little research on DWEP.

Lipid metabolism is essential in sustaining normal physiological activities in the organism [13]. Studies have shown that lipid metabolism is actively involved in tadpole metamorphosis [14]. Furthermore, research has established that fat serves as the primary energy source utilized by tadpoles during metamorphosis [14]. Thus, maintaining the liver’s healthy lipid metabolism encourages tadpole growth and metamorphosis. In addition, numerous studies have demonstrated that the gut microbiota can affect host fat metabolism via multiple mechanisms [15]. In an experiment involving the metamorphosis of bullfrog tadpoles, Zhu observed that thyroid powder stimulated the growth of *Akkermansia*, boosting the tadpoles’ fat metabolism and subsequently aiding their growth [16]. The variation in dietary components destabilizes the equilibrium of intestinal homeostasis in the host by reshaping the intestinal microbiota of fish and modifying metabolic processes and the abundance of essential symbiotic species [17]. Hence, an experiment was designed to assess and compare the impacts of DWEP and FM on the growth, lipid metabolism, antioxidative stress and intestinal flora of tadpoles. This study aimed to identify alternatives to FM, enhance tadpole metamorphosis and growth, advance bullfrog farming and aid in aquaculture nutrition and sustainable health management.

## 2. Materials and Methods

### 2.1. Experimental Feeds

DWEP was provided by XinruiSen Trading (Dalian, China) Co., Ltd. Feeds were formulated with poultry powder, soybean meal and soy protein concentrate as the primary protein sources and soybean oil as the primary lipid source. A basal diet without FM and DWEP was prepared as the CON group. Two gradients (5% and 10%) of DWEP and FM were separately added to formulate four experimental isonitrogenous diets with lipids. These diets were labeled E5, E10, F5 and F10, respectively (Table 1).

According to the formula, the feed ingredients were pre-mixed and then ground using a super-fine grinder and sifted through an 80-mesh sieve. Next, the treated ingredients were placed into a twin-screw diet extruder to pelletize and dried in the sunlight. Finally, the dried pellets were ground and sifted to create powdered feeds. The prepared feed was kept at −20 °C until the end of the breeding experiment.

### 2.2. Feeding Management

Feeding experiments were conducted at the bullfrog experimental bases of Hunan Agricultural University (Li Town, Li County, Changde City, Hunan Province and Changtang Town, Nanning, Guangxi Province, respectively). Before separation, the tadpoles were temporarily reared for 10 days in a net box (4.8 m × 2.7 m × 0.65 m) with a circulating water system to adapt to the experimental environment.

A total of five treatment groups were designed, with three replicates in each group. Healthy tadpoles (0.28 ± 0.01 g) were selected at the end of the staging period and placed into 15 experimental net boxes (0.8 m × 0.8 m × 0.6 m) at a density of 900 tails/m^2^ (n = 576), with an initial feeding rate of 6% of body weight, and fed three times a day (7:00, 12:00 and 17:00).

The feeding rate was adjusted every seven days based on the tadpoles’ feeding activity and the weekly average temperature. In addition, metamorphosed tadpoles were removed from the tank, and the feeding ratio was adjusted according to the weight of the remaining tadpoles. The bottom of the net box was cleaned with a siphon tube every 10 days to remove residual bait, feces and other pollutants. The physical and chemical properties of the water remained within the normal range during the experiment. The mean environmental temperature was 24.0 °C, the mean temperature of the water was 21.8 °C, the dissolved oxygen level was ≥4.0 mg/L, the pH ranged from 7.7 to 7.8, the ammoniacal nitrogen level was <0.4 mg/L and the nitrosative nitrogen level was <0.005 mg/L. The experiment lasted for 65 days.

### 2.3. Determination of Growth Indices

The tadpoles were classified into two categories according to their developmental status: the metamorphosed stage (after metamorphosis, possessing limbs and lacking a tail) and the pre-metamorphosed stage (before metamorphosis, possessing a tail). During the breeding experiment, the number of metamorphosed tadpoles in each cage was recorded every night after the emergence of the first metamorphosed tadpoles to calculate the cumulative metamorphosis number. At the end of the breeding experiment, the tadpoles (pre-metamorphosis) in each net box were weighed and counted. The number and weight of pre-metamorphosed tadpoles, as well as the amount of feed, were recorded and used to calculate growth indicators.

### 2.4. Ingredient Content and Liver Slices

The nutritional composition of the tadpoles’ whole liver and the feed was measured. The crude lipid content was measured using the Soxhlet extraction method, while the crude protein content was measured using the Kjeldahl method.

Nine tadpoles were randomly selected from each group, and the intact liver was excised on ice, promptly fixed in a paraformaldehyde solution for 24 h, rinsed in microfluidic water for 6 h, subsequently dehydrated with varying gradients of alcohol, rendered transparent with xylene and then subjected to transdermal waxing, embedding, slicing and staining to generate liver tissue sections. The aforementioned parts were scanned, subsequently examined and analyzed utilizing the Slide Viewer 2.9.0 image analysis software.

### 2.5. Hepatic Lipid Metabolism and Antioxidant Biochemical Indicators

Twenty-four tadpoles were randomly selected from each group, and their livers were excised on ice and deposited in enzyme-free tubes and then preserved at −80 °C. Twelve liver samples from each group were collected to measure the following indicators: total cholesterol (TC), triglyceride (TG), lipoprotein lipase (LPL), hepatic lipase (HL), lipase (LPS), total antioxidant capacity (T-AOC), superoxide dismutase (SOD), catalase (CAT), glutathione (GSH), glutathione peroxidase (GPX) and malondialdehyde (MDA). The test kits were purchased from the Nanjing Jiancheng Bioengineering Institute (Nanjing, China).

Twelve additional liver samples from each group were analyzed for liver gene expression. RNA was extracted using a Trizol kit, and, after assessing its quality by agarose gel electrophoresis and determining the concentration, a reverse transcription reaction was performed to synthesize cDNA. Beta-actin served as an internal reference gene. The QPCR primers used in this experiment are shown in Table 2. Annex 1 details the relevant program configurations and reaction systems.

### 2.6. Determination of Intestinal Flora

The bacterial community DNA was sequenced in two parts using the Illumina NovaSeq machine. The raw sequence data were decoded and processed with the demux plugin, followed by quality filtering, denoising, splicing and chimera removal using the DADA2 plugin. The aforementioned sequences underwent 100% sequence similarity merging to produce the feature sequence amplicon sequence variation and abundance data table.

### 2.7. Data Processing

In this experiment, data analysis methods were selected based on Shapiro–Wilk normality tests and Levene’s homogeneity of variance tests. To compare the CON group with the FM or DWEP group, a one-way ANOVA or Kruskal–Wallis test was used. Independent-sample *t*-tests were used to compare the F5 and E5 groups or the F10 and E10 groups.

Data were expressed as the mean ± standard error (mean ± SE). Multiple comparisons were conducted utilizing Duncan’s technique or Dunn’s method, with statistically significant differences identified at a *p*-value of 0.05. Statistical analyses were conducted with the SPSS 26.0 software (IBM, New York, NY, USA). Image rendering was performed utilizing Python programming.

## 3. Results

### 3.1. Growth Data of Tadpoles

Compared with the CON group, the mean weight of BM, weight gain rate of BM and metamorphosis rate of tadpoles were significantly higher (*p* < 0.05) and the feed conversion ratio was significantly lower (*p* < 0.05) in the FM group (F5, F10) and DWEP group (E5, E10). In the FM group, there were no significant differences observed in the mean weight of BM, weight gain rate of BM and feed conversion ratio compared with the DWEP group (*p* > 0.05). However, the metamorphosis rate in the E5 group was significantly lower than that in the F5 group (*p* < 0.05) (Table 3).

**Figure 1:**
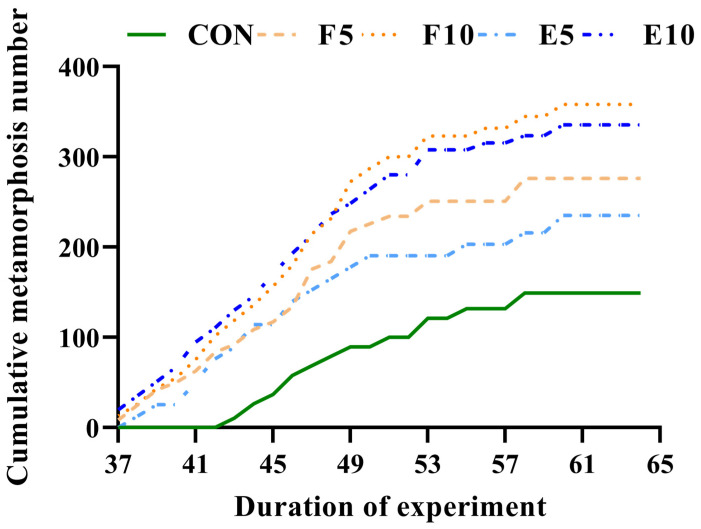
The cumulative metamorphosis numbers. Note: Starting from the 37th day of the culture experiment, the metamorphosed tadpoles from all nets were counted every night.

From Figure 1, it can be observed that FM and DWEP treatment promoted the tadpole metamorphosis process. The tadpoles in the FM and DWEP groups entered the metamorphosed stage on the 37th day after the start of the experiment, whereas this occurred on the 43rd day in the CON group. After 43 days, the cumulative metamorphosis numbers of the CON, FM and DWEP groups increased with the progression of the experiment. After the 58th day of the experiment, the cumulative metamorphosis numbers of the CON and F5 groups essentially no longer changed. In contrast, the cumulative metamorphosis numbers of the E5, E10 and F10 groups continuously increased until the 60th day of the experiment. Throughout the process, the highest cumulative metamorphosis numbers were obtained in the FM, group followed by the DWEP group and CON group.

### 3.2. Hepatic Crude Fat Content of Tadpoles and the Structure of the Liver

**Figure 2:**
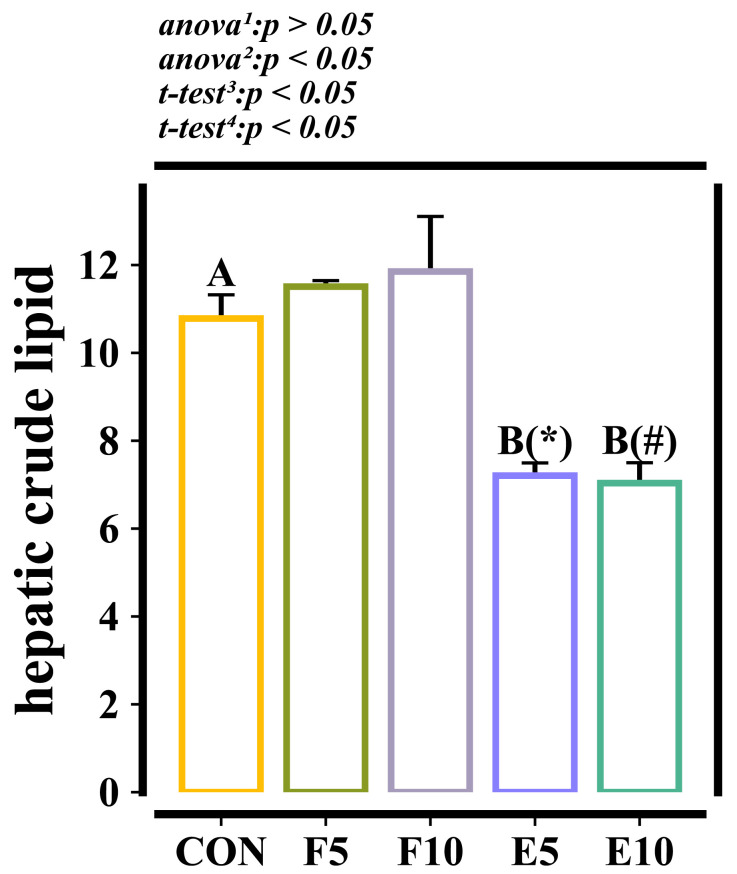
Hepatic crude fat content of tadpoles (wet weight, %). Note: The different capital letters indicate significant differences between the CON group and DWEP group (*p* < 0.05). The * in the E5 group indicates significant differences compared with the F5 group (*p* < 0.05). The # in the E10 group indicates significant differences compared with the F10 group (*p* < 0.05). ^1,2^: The *p*-value of the one-way ANOVA or Kruskal–Wallis test for the CON group compared to the FM group or DWEP group, respectively. ^3,4^: The *p*-value of the independent-samples *t*-test for groups E5 and F5, as well as groups E10 and F10.

Adding FM did not affect the crude lipid content in the livers of the tadpoles (*p* > 0.05), but it exhibited a significant decrease in the DWEP group compared to the CON and FM groups (*p* < 0.05) (Figure 2).

**Figure 3:**
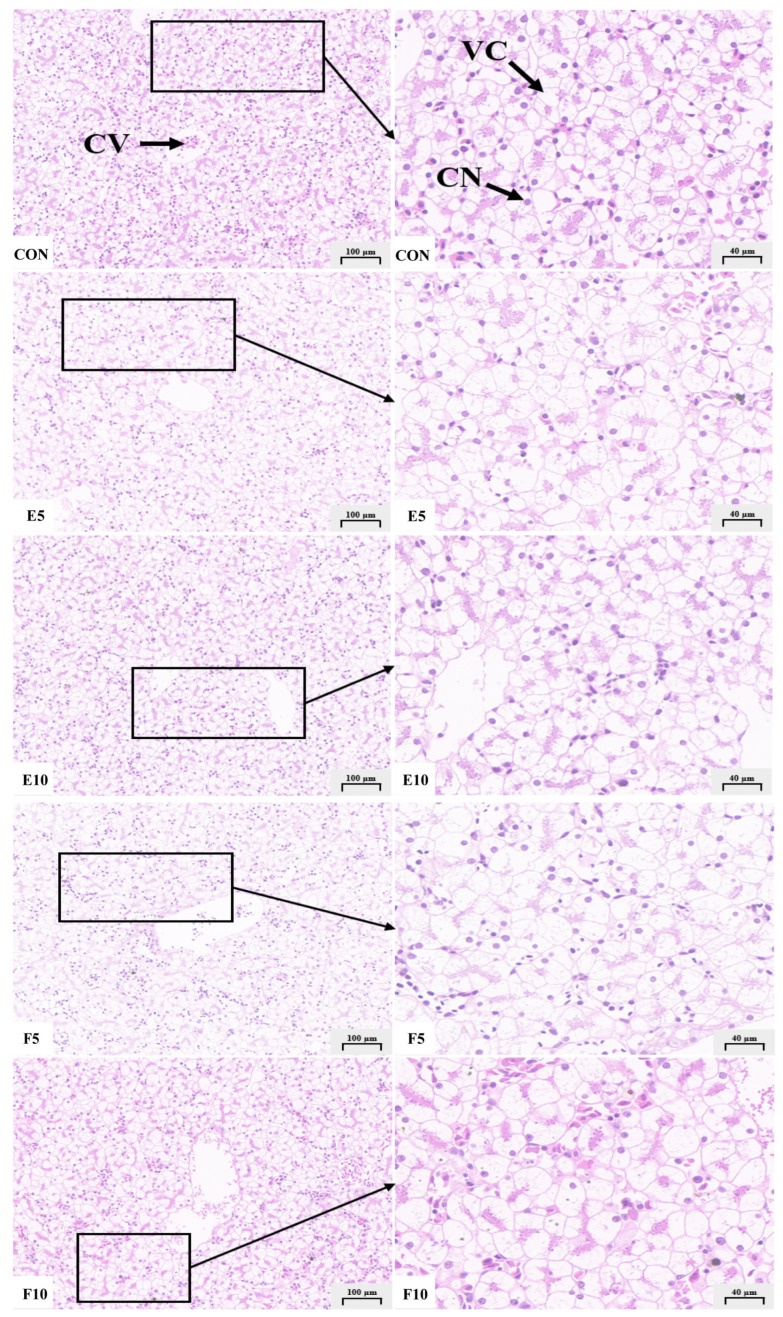
Liver slice (100× and 500×). Note: CV, central veins (the veins in the center of the hepatic lobule are surrounded by radial liver cells, which are important vessels for the exchange of substances between the liver and other organs); CN, cell nucleus; VC, vacuolization.

The observation and analysis of the hepatocytes around the central vein of the tadpole liver via a microscope and the Image J 1.8.0_345 software showed that DWEP and FM inclusion did not affect the size and structural integrity of the tadpoles’ hepatocytes. The addition of DWEP to the feed reduced the vacuolization of the hepatocytes compared with FM (Figure 3).

### 3.3. Biochemical Indicators Related to Lipid Metabolism in the Liver

**Figure 4:**
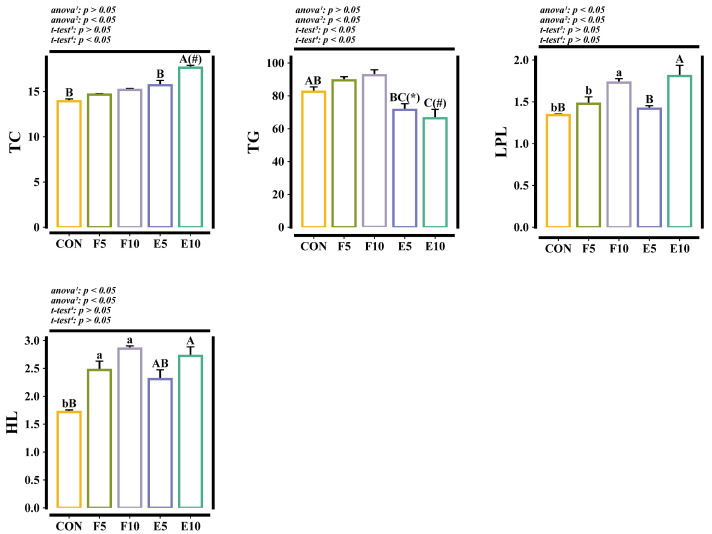
Biochemical indicators related to lipid metabolism in liver. Note: The different lowercase letters indicate significant differences between the CON group and FM group (*p* < 0.05). The different capital letters indicate significant differences between the CON group and DWEP group (*p* < 0.05). The * in the E5 group indicates significant differences compared with the F5 group (*p* < 0.05). The # in the E10 group indicates significant differences compared with the F10 group (*p* < 0.05). ^1,2^: The *p*-value of the one-way ANOVA or Kruskal–Wallis test for the CON group compared to the FM group or DWEP group, respectively. ^3,4^: The *p*-value of the independent-samples *t*-test for groups E5 and F5, as well as groups E10 and F10. TC, total cholesterol (mmol/g prot × 10^2^); TG, triglyceride (mmol/g prot × 10^2^); LPL, lipoprotein lipase (U/mg prot); HL, hepatic lipase (U/mg prot).

In comparison to the CON group, the hepatic LPL and HL levels in the FM and DWEP groups were significantly increased (*p* < 0.05), but no significant difference was seen between the FM and DWEP groups (*p* > 0.05). In the CON group, the hepatic TG content showed no significant difference compared with the FM group (*p* > 0.05), and it was lower in the DWEP group (*p* < 0.05); here, it was also lower than that in the FM group (*p* < 0.05). In addition, DWEP treatment distinctly increased the hepatic TC content (*p* < 0.05), while no significant differences were observed between the CON and FM groups (*p* > 0.05) (Figure 4).

### 3.4. Relative Expression Levels of Hepatic Genes

**Figure 5:**
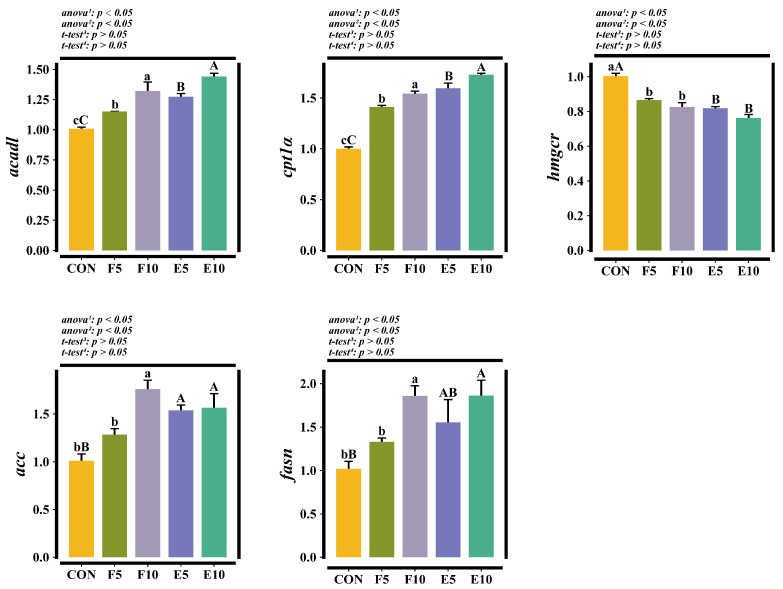
Relative expression levels of hepatic genes. Note: The different lowercase letters indicate significant differences between the CON group and FM group (*p* < 0.05). The different capital letters indicate significant differences between the CON group and DWEP group (*p* < 0.05). ^1,2^: The *p*-value of the one-way ANOVA or Kruskal–Wallis test for the CON group compared to the FM group or DWEP group, respectively. ^3,4^: The *p*-value of the independent-samples *t*-test for groups E5 and F5, as well as groups E10 and F10. *acadl*, long-chain-acyl-CoA dehydrogenase; *cpt1α*, carnitine palmitoyltransferase 1α; *hmgcr*, 3-hydroxy-3-methylglutaryl-CoA reductase; *acc*, acetyl-CoA carboxylase; *fasn*, fatty acid synthase.

Compared with the CON group, the relative expression levels of hepatic *acadl*, *acc*, *cpt1α* and *fasn* were clearly upregulated (*p* < 0.05) and that of hepatic *hmgcr* was significantly downregulated in the FM and DWEP groups (*p* < 0.05). There was no significant difference observed in the above-mentioned genes’ abundance between the FM and DWEP groups (*p* > 0.05) (Figure 5).

### 3.5. Enzyme Activity Related to Antioxidant Capacity in the Liver

**Figure 6:**
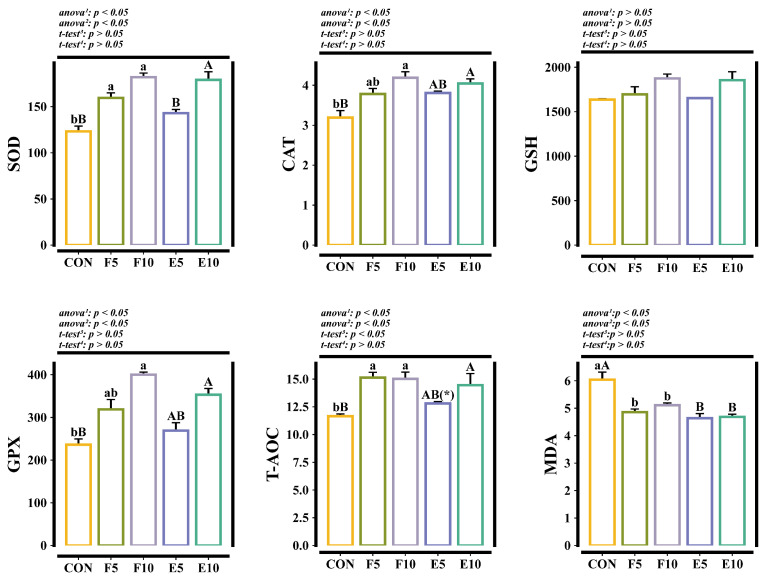
Enzyme activity related to antioxidant capacity in liver. Note: The different lowercase letters indicate significant differences between the CON group and FM group (*p* < 0.05). The different capital letters indicate significant differences between the CON group and DWEP group (*p* < 0.05). The * in the E5 group indicates significant differences compared with the F5 group (*p* < 0.05). ^1,2^: The *p*-value of the one-way ANOVA or Kruskal–Wallis test for the CON group compared to the FM group or DWEP group, respectively. ^3,4^: The *p*-value of the independent-samples *t*-test for groups E5 and F5, as well as groups E10 and F10. SOD, superoxide dismutase (U/mg prot); CAT, catalase (U/mgprot); GSH, glutathione (μmol/g prot); GPX, glutathione peroxidase (U); T-AOC, total antioxidant capacity (mmol/g prot × 10^2^); MDA, malondialdehyde (nmol/mg prot).

Compared with the CON group, there was no significant difference in hepatic GSH between the FM and DWEP groups (*p* > 0.05). FM and DWEP addition significantly decreased the hepatic MDA content (*p* < 0.05) and enhanced the hepatic SOD, CAT, GPX and T-AOC activity (*p* < 0.05). Compared with the FM group, there was no significant difference in the hepatic SOD, CAT, GSH, GPX and MDA in the DWEP group (*p* > 0.05). The hepatic T-AOC in the E5 group was significantly lower than that in the F5 group (*p* < 0.05) (Figure 6).

### 3.6. Gut Microbial Community Alteration

**Figure 7:**
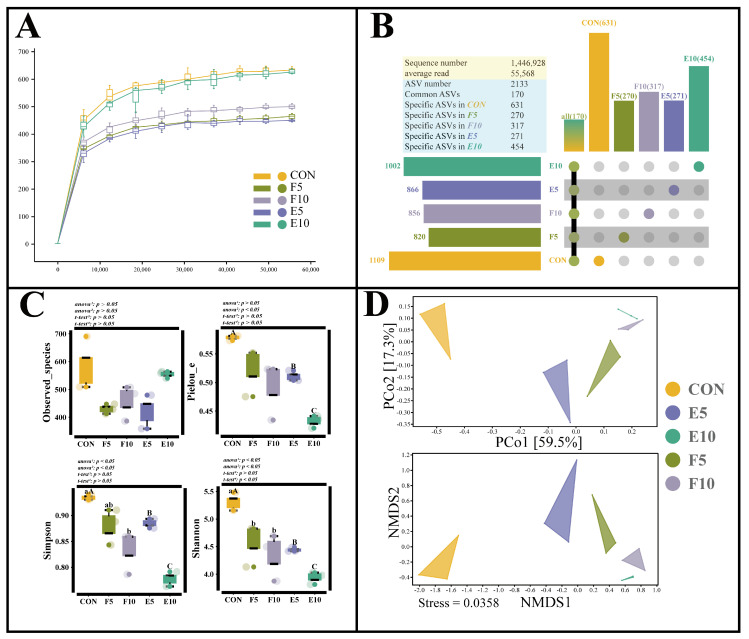
Gut microbial diversity analysis. Note: The different lowercase letters indicate significant differences between the CON group and FM group (*p* < 0.05). The different capital letters indicate significant differences between the CON group and DWEP group (*p* < 0.05). ^1,2^: The *p*-value of the one-way ANOVA or Kruskal–Wallis test for the CON group compared to the FM group or DWEP group, respectively. ^3,4^: The *p*-value of the independent-samples *t*-test for groups E5 and F5, as well as groups E10 and F10. (**A**) Rarefaction curves (Chao 1) of each treatment group; (**B**) specific and shared ASVs among treatment groups; (**C**) microbial community α−diversity index (Observed_species, Pielou_e, Simpson and Shannon); (**D**) microbial community β−diversity index (principal coordinate analysis and non-metric multidimensional scaling).

Observing Figure 7A, it is noted that, as the sampling depth increases, the curves for each group exhibit a tendency to flatten, signifying that the sequencing results effectively capture the diversity inherent in the experimental data. A total of 14,469,280 high-quality sequences were obtained through 16S rRNA sequencing, resulting in an average of 55,568 reads per sample. Across all samples, 2133 bacterial amplicon sequence variants (ASVs) were identified, with 170 ASVs shared among all groups. The CON group, F5 group, F10 group, E5 group and E10 group contained 1109, 820, 856, 866 and 631 ASVs, respectively. Each group had unique ASVs, with counts of 631, 270, 317, 271 and 454 for the CON, F5, F10, E5 and E10 groups, respectively (Figure 7B).

When compared to the CON group, the α-diversity metrics (including Observed_species, Pielou_e, Simpson and Shannon) of the gut microbiota in the FM and DWEP groups exhibited a decline. Specifically, the DWEP group experienced a notable decrease in the Pielou_e, Simpson and Shannon values (*p* < 0.05), while the FM group exhibited a significant drop in the Shannon value (*p* < 0.05). This suggests that the α-diversity of the gut microbial communities in these two groups was diminished. However, when comparing the FM and DWEP groups, the latter did not show significant changes in these metrics (*p* > 0.05) (Figure 7C). Furthermore, Figure 7D provides additional evidence that the inclusion of DWEP and FM in the diet significantly altered the microbial community structure.

**Figure 8:**
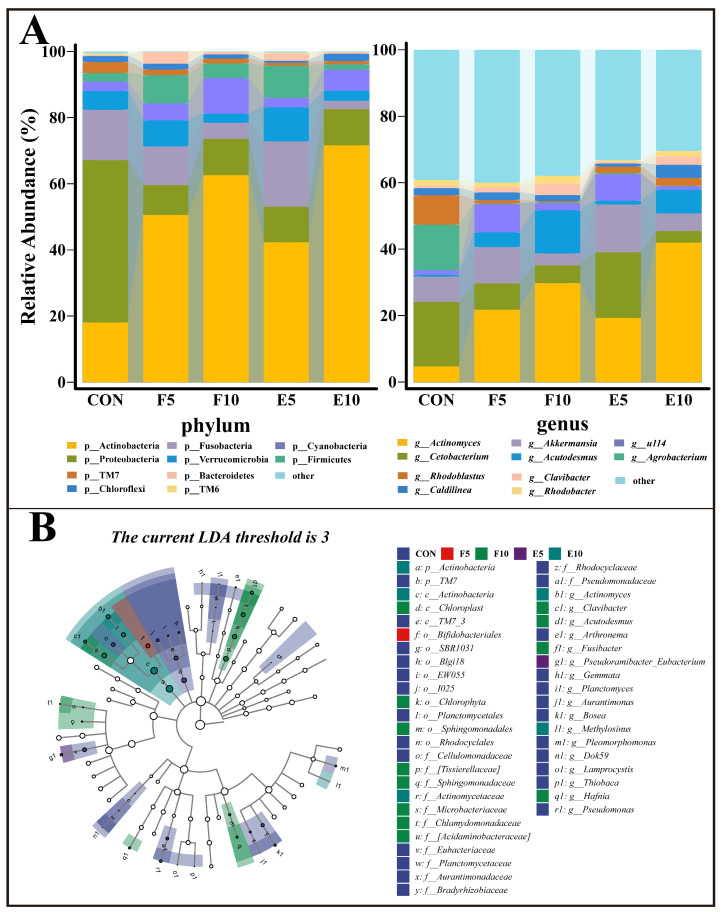
Microbial composition and biomarkers. Note: (**A**) Composition of the gut microbial community (phylum and genus); (**B**) LDA effect size analysis. The rings represent the species, genus, family, order, class and phylum from outside to inside. The species with an LDA score > 3 were defined as statistically different biomarkers.

At the phylum level, the top three dominant phyla in the intestinal flora of the tadpoles were Actinobacteria, Proteobacteria and Fusobacteriota. Relative to the CON group, there was an increase in the relative abundance of Actinobacteria in both the FM and DWEP groups, whereas the relative abundance of Proteobacteria decreased. Moreover, Fusobacteria had the lowest relative abundance in the F10 and E10 groups, at 4.85% and 2.51%, respectively. At the genus level, the genera with the highest relative abundance were *Actinomyces*, *Cetobacterium* and *Akkermansia*. *Actinomyces* is the main genus in the phylum Actinobacteria, and its trend is consistent with that of the phylum Actinobacteria. In addition, the highest abundance of intestinal *Akkermansia* was found in group E5 (10.18%) and group F5 (7.72%) (Figure 8A). The Lefse analysis revealed that Actinobacteria and *Actinomyces* could serve as biomarkers to differentiate the E10 group from other groups, with an LDA score of 3 (Figure 8B).

**Figure 9:**
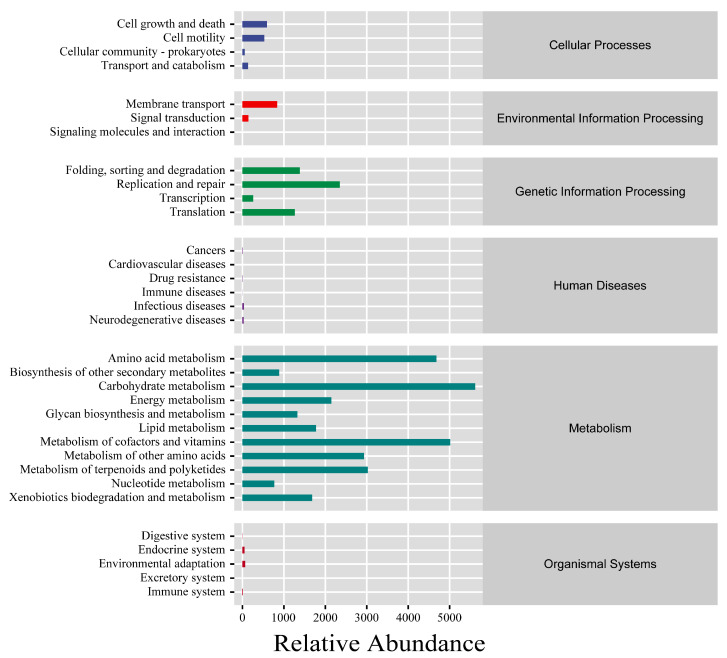
Statistics of microbial metabolic pathways.

In this study, the metabolic pathway analysis revealed that the main metabolic pathways of intestinal microorganisms are concentrated in metabolic functions, including amino acid metabolism (4682.17), carbohydrate metabolism (5619.01) and lipid metabolism (1774.54) (Figure 9).

## 4. Discussion

### 4.1. Effects of Different Levels of FM and DWEP on the Growth Performance of Tadpoles

Growth performance serves as a dependable indicator in assessing the efficacy of feed in aquaculture. Dietary FM inclusion improved the growth performance of tadpoles, which might be linked to FM’s higher nutrient assimilation efficiency relative to soy protein concentrate (SPC). Although SPC is a plant-derived protein source, its utilization may be hindered by anti-nutritional factors [18]. Conversely, FM not only delivers a comprehensive profile of essential amino acids but also minimizes such inhibitory effects, potentially enhancing nutrient utilization. Similarly to the FM group, the weight gain rate and feed conversion rate of the tadpoles in the DWEP group were significantly superior to those in the CON group. This aligns with the findings obtained after incorporating egg powder into broiler feed [19]. It could be related to the high content of cholesterol, as well as lecithin, in DWEP. Cholesterol and lecithin are integral parts of cell membranes and are essential for cell growth [20,21]. In addition, lecithin emulsifies lipids, promotes fat absorption and improves the efficiency of energy utilization [22]. Notably, the soybean oil levels were adjusted downward in the DWEP group to achieve formulation equilibrium. While soybean oil predominantly serves as a triglyceride-based energy source, it is inherently limited in bioactive lipid constituents that support metabolic functions [23]. Furthermore, it is widely recognized that the accumulation of more nutrients in tadpoles’ bodies prior to metamorphosis (higher mean weight of BM) would be beneficial for their subsequent growth.

The metamorphosis rate of tadpoles is a point of focus in the farming of Lithobates catesbeianus. The metamorphosis process not only involves changes in the external morphology but also the restructuring of the internal organs and the adjustment of physiological functions [24]. It is mainly regulated by endogenous thyroid hormones [25]. The results of this experiment showed that both FM and DWEP significantly promoted the metamorphosis of tadpoles, which may be related to their high content of vitamin D [26,27] and cholesterol. Vitamin D plays a critical role in regulating the physiological functions of multiple organ systems, including the thyroid gland, through its involvement in hormonal homeostasis and immune modulation [28]. Several studies have indicated that the supplementation of vitamin D in animal models of thyroiditis or hypothyroidism enhances thyroid function [29]. Cholesterol functions as a precursor for corticosteroid production [30]. Research has indicated that corticosteroids could boost the binding affinity of thyroid hormone T3 in the tadpole’s tail and elevate the tissue sensitivity to thyroid hormones [25]. In addition, corticosteroids were able to enhance the activity of 5′-deiodinase [31], one of the enzymes that determines the concentrations of active thyroid hormones within tadpole cells [32]. However, the precise mechanism underlying the improved metamorphosis rate remains to be further explored comprehensively.

### 4.2. Effects of Different Levels of FM and DWEP on the Lipid Metabolism of Tadpoles

LPL and HL are two essential enzymes that hydrolyze triglycerides from chylomicrons into free fatty acids [33]. The body’s utilization of free fatty acids involves two primary processes: the de novo lipogenesis and β-oxidation of fatty acids [34]. In a normal physiological state, the two processes are usually in a dynamic balance, flexibly regulating fatty acid synthesis and catabolism to maintain cellular energy metabolism. *Acc* and *fasn* serve as key enzymes for the de novo lipogenesis of fatty acids [10,35], while *cpt1α* acts as the rate-limiting enzyme for fatty acid β-oxidation [36], with *acadl* responsible for the initial phase of this process [37]. The results of this experiment indicated that both FM and DWEP activated lipid metabolism at the transcriptional level in the tadpoles’ livers. *Hmgcr* is a vital rate-limiting enzyme in cholesterol production [38]. In this experiment, FM and DWEP downregulated *hmgcr* expression, perhaps due to the certain amounts of cholesterol in FM and DWEP, which suppressed the cholesterol biosynthesis pathway. Comparable outcomes have been noted in turbot (*Scophthalmus maximus*) [39]. Furthermore, it was observed that the crude fat and hepatic TG remained unchanged following the incorporation of FM into the feed. On the other hand, hepatic fat accumulation after DWEP inclusion was significantly reduced, potentially due to its high lecithin content. The phospholipids and choline in lecithin could improve mitochondrial function, promoting fat oxidation [40]. Lecithin is also involved in lipoprotein synthesis, the emulsification of lipids and the stabilization of lipoproteins, thereby aiding the transfer of triglycerides from the liver [41].

### 4.3. Effects of Different Levels of FM and DWEP on the Antioxidant Properties of Tadpoles

Excessive free radicals are the root cause of oxidative damage in animal organisms. Typically, the body maintains an equilibrium between the generation and elimination of free radicals. However, when this equilibrium is disrupted, the body experiences oxidative harm. Excessive free radicals can induce detrimental alterations to cellular constituents, including proteins, lipids and DNA, potentially resulting in cellular damage or mortality [42]. The major enzymes SOD, CAT and GPX, as well as the vital antioxidant molecule GSH, are essential components of the body’s antioxidant system [43]. The extent of oxidative damage is effectively reduced by the synergistic action of these components through scavenging intracellular oxygen radicals and reducing the production of lipid peroxidation products (MDA, etc.) [44]. Furthermore, T-AOC, serving as a comprehensive measure of the body’s overall ability to withstand oxidative stress, is crucial in evaluating the oxidative condition of cells and tissues [45]. The results indicated that both FM and DWEP effectively enhanced the antioxidant capacity and decreased oxidative damage in the tadpoles. Research has indicated that DHA and EPA, which are abundant in FM, could boost the activity of antioxidant-related enzymes, therefore accelerating the elimination of free radicals [46]. Furthermore, EPA and DHA enhanced the fluidity and structural integrity of cell membranes, particularly in mitochondria, diminishing free radical generation [46]. In addition, lecithin, abundant in DWEP and possessing double bonds, exhibited resistance to oxidation and had the potential to alleviate oxidative damage upon metabolism in the body [47]. DWEP also contains other antioxidant components, such as phosvitin, ovotransferrin and carotenoids, which can chelate divalent metal cations, inhibit free radical formation and stimulate the synthesis of antioxidant enzymes [48,49].

### 4.4. Effects of Different Levels of FM and DWEP on the Intestinal Flora of Tadpoles

Microbial metabolites, including short-chain fatty acids (SCFAs) and bile acids, function both as metabolic substrates and signaling molecules, intricately linked to body fat metabolism via the activation of signaling pathways like PPARγ and FXR, or through binding to specific receptors [50]. In this experiment, the primary function of gut microorganisms was associated with metabolic pathways, which encompass lipid metabolism. Additionally, the inclusion of DWEP or FM led to a substantial rise in the relative abundance of Actinobacteria and *Actinomyces*, particularly in the E10 group, where they served as biomarkers, and also resulted in a notable decline in the relative abundance of Proteobacteria. *Actinomyces’* secondary metabolites are a significant reservoir of antibiotics. Their higher concentrations in the FM and DWEP groups enhanced antibiotic synthesis in the intestine and prevented the survival of harmful bacteria, potentially resulting in a reduction in the α-diversity in these groups [17]. Proteobacteria are a group of Gram-negative bacteria that include lipopolysaccharides in their cell walls. These lipopolysaccharides have a tendency to cause inflammatory reactions in animals [51]. The overproliferation of Proteobacteria may be associated with health problems such as intestinal inflammation and lipid metabolism disorder. Moreover, the addition of 5% FM or DWEP to the feed increased the abundance of *Akkermansia*, whereas the addition of 10% FM or DWEP reduced the abundance of Fusobacteria. *Akkermansia* is closely linked to the host’s metabolic and immunological responses and can mitigate intestinal inflammation [52]. In addition, *Akkermansia* synthesizes SCFAs along with additional metabolites, which can affect the metabolic equilibrium of glucose and lipid metabolism [53]. Fusobacteria encompass a diverse range of pathogenic bacteria that have the capability to induce oxidative stress within the body [54]. Hence, the inclusion of DWEP or FM in the diet resulted in an increase in the ratio of positive microbes and a reduction in the ratio of unfavorable microbes.

## 5. Conclusions

This experiment demonstrated that the feeding of FM or DWEP facilitated tadpole growth, elevated the metamorphosis rates, stimulated fat metabolism, enhanced the antioxidant capacity and encouraged the proliferation of advantageous microbes, while reducing the presence of detrimental bacteria. Thus, DWEP can serve as a feasible substitute for FM in production.

## Figures and Tables

**Table 1:** Compositions and nutrient levels of the experimental diets (dry matter %).

Ingredient (%)	CON *	F5 *	F10 *	E5 *	E10 *
FM	0	5	10	0	0
DWEP	0	0	0	5	10
Poultry powder	12	12	12	12	12
Soybean meal	15	15	15	15	15
Soy protein concentrate	32.71	27.6	22.45	28.69	25.4
Microcrystalline cellulose	0	0.43	0.9	0.98	1.24
Rice bran meal	6	6	6	6	6
Flour	26	26	26	26	26
Soybean oil	4.24	3.92	3.6	2.28	0.31
Premix ^1^	2	2	2	2	2
Monocalcium phosphate	1.5	1.5	1.5	1.5	1.5
Choline chloride	0.5	0.5	0.5	0.5	0.5
Antioxidant	0.02	0.02	0.02	0.02	0.02
Mold inhibitor	0.03	0.03	0.03	0.03	0.03
Total	100	100	100	100	100
Crude protein	43.51	43.49	42.87	42.73	43.64
Crude fat	7.27	7.83	7.48	7.35	7.24

* CON: 0% DWEP and FM; E5: 5% DWEP; E10: 10% DWEP; F5: 5% FM; F10: 10% FM. ^1^ The premix per kg of diet: KCl 200 mg, KI (1%) 60 mg, CoCl_2_·6H_2_O (1%) 50 mg, CuSO_4_·5H_2_O 30 mg, FeSO_4_·H_2_O 400 mg, ZnSO_4_·H_2_O 400 mg, MnSO_4_·H_2_O 150 mg, Na_2_SeO_3_·5H_2_O (1%) 65 mg, MgSO_4_·H_2_O 2000 mg, zeolite power 3645.85 mg, VB1 12 mg, riboflavin 12 mg, VB6 8 mg, VB12 0.05 mg, VK3 8 mg, inositol 100 mg, pantothenic acid 40 mg, niacin acid 50 mg, folic acid 5 mg, biotin 0.8 mg, VA 25 mg, VD 35 mg, VE 50 mg, VC 100 mg, ethoxyquin 150 mg, flour 2 434.15 mg.

**Table 2:** Primers used in the experiment.

Gene	GenBank Number ^1^	Forward Primer (5′-3′)	Reverse Primer (5′-3′)	Amplification Efficiency (%)
*fasn*	LH228595.1	CCTCCACGCCAGAACAAGAT	GATATTTTTATGAGTGGACATTGTATCGA	99.28
*acc*	LH212450.1	GTTAAAGCTGCCATCCTCACTGT	TGTCCGTCTGGCTAAGATGGT	93.66
*acadl*	LH364687.1	TGAGGAAACCCGGAACTATGTC	TGTGCTGCACGGTCTGTAAGT	92.73
*cpt1α*	LH022414.1	TGATTGGCAAAATCAAAGAACATC	AATGCTCTGACCCTGGTGAGA	95.72
*hmgcr*	LH363056.1	TGCATCCTCAAAAACCCAGAT	GGGATGTGTTTAGCATTCACCAA	91.53
*beta actin*	AB094353	ATGATGCTCCTCGTGCTGTGT	CCCCATTCCAACCATGACA	94.54

^1^ The GenBank number comes from the National Center for Biotechnology Information (NCBI).

**Table 3:** Effects of different levels of FM and DWEP on the growth performance of tadpoles.

Item	CON	F5	F10	E5	E10
initial average weight/g	0.28 ± 0.00	0.28 ± 0.00	0.28 ± 0.00	0.28 ± 0.01	0.28 ± 0.00
^1^ mean weight of BM/g	3.72 ± 0.04 ^cC^	4.25 ± 0.01 ^b^	4.58 ± 0.02 ^a^	4.18 ± 0.08 ^B^	4.48 ± 0.03 ^A^
^2^ weight gain rate of BM/%	1228.03 ± 14.29 ^cC^	1416.97 ± 1.85 ^b^	1534.30 ± 7.52 ^a^	1392.06 ± 29.89 ^B^	1501.59 ± 11.97 ^A^
^3^ metamorphosis rate/%	25.92 ± 2.83 ^cC^	47.92 ± 2.41 ^b^	62.16 ± 6.68 ^a^	40.80 ± 2.82 ^B(^*^)^	58.22 ± 0.60 ^A^
^4^ feed conversion rate	1.37 ± 0.05 ^aA^	1.19 ± 0.00 ^b^	1.06 ± 0.01 ^c^	1.21 ± 0.00 ^B^	1.11 ± 0.01 ^C^
^5^ survival rate/%	98.22 ± 0.38	97.83 ± 0.06	98.49 ± 0.41	98.42 ± 0.28	97.32 ± 0.11

Note: The different lowercase letters indicate significant differences between the CON group and FM group (*p* < 0.05). The different capital letters indicate significant differences between the CON group and DWEP group (*p* < 0.05). The * in the E5 group indicates significant differences compared with the F5 group (*p* < 0.05). ^1^ Mean weight before metamorphosis (BM) (g) = total weight of pre-metamorphosed tadpoles/total number of pre-metamorphosed tadpoles × 100; ^2^ weight gain rate of BM (%) = (total final pre-metamorphosed tadpole weight—total initial corresponding tadpole weight)/total initial corresponding tadpole weight × 100; ^3^ metamorphosis rate (%) = metamorphosed tadpole count/initial tadpole count × 100; ^4^ feed conversion rate = feed weight/final total tadpole weight; ^5^ survival rate (%) = final number of tadpoles/initial number of tadpoles × 100.

## Data Availability

The data of this study are available from the corresponding author upon reasonable request.
